# Multivariate piecewise linear regression model to predict radiosensitivity using the association with the genome-wide copy number variation

**DOI:** 10.3389/fonc.2023.1154222

**Published:** 2023-10-02

**Authors:** Joanna Tobiasz, Najla Al-Harbi, Sara Bin Judia, Salma Majid Wakil, Joanna Polanska, Ghazi Alsbeih

**Affiliations:** ^1^ Department of Data Science and Engineering, Silesian University of Technology, Gliwice, Poland; ^2^ Department of Computer Graphics, Vision and Digital Systems, Silesian University of Technology, Gliwice, Poland; ^3^ Radiation Biology Section, Biomedical Physics Department, King Faisal Specialist Hospital and Research Centre, Riyadh, Saudi Arabia; ^4^ Department of Genetics, King Faisal Specialist Hospital and Research Centre, Riyadh, Saudi Arabia; ^5^ Laboratory of Neurogenetics, National Institutes of Health, Rockville, MD, United States

**Keywords:** radiosensitivity, surviving fraction at 2 Gy (SF2), dynamic programming, linear regression, Affymetrix CytoScan HD microarrays, copy number variation (CNV), radiogenomics

## Abstract

**Introduction:**

The search for biomarkers to predict radiosensitivity is important not only to individualize radiotherapy of cancer patients but also to forecast radiation exposure risks. The aim of this study was to devise a machine-learning method to stratify radiosensitivity and to investigate its association with genome-wide copy number variations (CNVs) as markers of sensitivity to ionizing radiation.

**Methods:**

We used the Affymetrix CytoScan HD microarrays to survey common CNVs in 129 fibroblast cell strains. Radiosensitivity was measured by the surviving fraction at 2 Gy (SF2). We applied a dynamic programming (DP) algorithm to create a piecewise (segmented) multivariate linear regression model predicting SF2 and to identify SF2 segment-related distinctive CNVs.

**Results:**

SF2 ranged between 0.1384 and 0.4860 (mean=0.3273 The DP algorithm provided optimal segmentation by defining batches of radio-sensitive (RS), normally-sensitive (NS), and radio-resistant (RR) responders. The weighted mean relative errors (MRE) decreased with increasing the segments' number. The borders of the utmost segments have stabilized after partitioning SF2 into 5 subranges.

**Discussion:**

The 5-segment model associated C-3SFBP marker with the most-RS and C-7IUVU marker with the most-RR cell strains. Both markers were mapped to gene regions (MCC and SLC1A6, respectively). In addition, C-3SFBP marker is also located in enhancer and multiple binding motifs. Moreover, for most CNVs significantly correlated with SF2, the radiosensitivity increased with the copy-number decrease.

In conclusion, the DP-based piecewise multivariate linear regression method helps narrow the set of CNV markers from the whole radiosensitivity range to the smaller intervals of interest. Notably, SF2 partitioning not only improves the SF2 estimation but also provides distinctive markers. Ultimately, segment-related markers can be used, potentially with tissues’ specific factors or other clinical data, to identify radiotherapy patients who are most RS and require reduced doses to avoid complications and the most RR eligible for dose escalation to improve outcomes.

## 1 Introduction

Individual variation in radiosensitivity is well recognized in clinical, epidemiological, and laboratory investigations and is largely attributed to genetic factors (1, 2). The genomic basis of radiosensitivity (radiogenomics) has a potential impact on personalized medicine, both in the treatment of cancer patients, where normal tissues and tumors differ in their response to radiotherapy, and in cancer predisposition, where exposure in occupational, diagnostic radiology, environmental and space radiation exposures may have different carcinogenic susceptibilities in populations (3, 4). Currently, the term radiogenomics is used ambiguously to refer to genetic variation associated with response to radiation (Radiation Genomics) or to cancer imaging features attributed to gene expression profiling (Imaging Genomics).

An important step in the development of radiogenomics is to devise statistical methods and algorithms that are capable of identifying a few important genetic variations between millions generated in genome-wide association studies (GWAS). Using twin studies, we reported that certain SNPs and their transcriptomic influence are associated with individual radiation sensitivity with a heritability estimate of 66% (5). In a previous study, an association between individual radiosensitivity, measured *in vitro* with clonogenic survival, and certain genetic polymorphic variations has been described that showed an increasing effect with an increasing number of the identified risk alleles (6). Based on these observations, a GWAS was initiated using the Affymetrix CytoScan HD microarrays that also enable the quantification of the copy number variation (CNV) in the genome. CNV is a type of structural variation where a stretch of DNA experiences gains (CNV>2) or losses (CNV<2) compared to the normal two copies (CNV=2), which affects gene dosage (7). Pilot analysis underlined the inadequacy and limitation of relying on the threshold value of mean radiosensitivity measurement between radio-sensitive (RS) and normally sensitive (NS) cell strains, which appeared as theoretical and conventional (8). Here we extend this study using a new strategy of combining qualitative and quantitative statistical approaches to identify potential biomarkers, taking into consideration not only the overall correlation between the radiosensitivity measure and CNVs but also its differences between RS, NS and radio-resistant (RR) cell strains.

The radiosensitivity of cell strains in culture is frequently characterized by the surviving fraction at 2 Gy (SF2) radiation dose assessed in a clonogenic survival assay (6). It represents the proportion of cells that maintain their ability to grow into colonies after the dose of 2 Gy irradiation. SF2 is considered a gold standard for measuring cell radiosensitivity, robust enough to be widely used in various radiation research projects (9). In many studies, SF2 is a base for grouping cell strains of similar sensitivity: usually denoted as RS and NS responders. In that case, the mean (6) or median (10, 11) SF2 value serves as a threshold since the SF2 distribution is approximately normal. The two obtained groups varying in radiosensitivity are being statistically compared in terms of features of interest like gene expression. However, the mean- or median-based division may result in the misassignment of cell strains of similar SF2 values surrounding the threshold to the opposite categories. This may lead to the loss of some significant information because cell strains close in their sensitivity can be tested against each other. The solution would be to stratify SF2 by extracting only cell strains that are unambiguously RS or RR and compare them while discarding all cell strains that are intermediate in their response to radiation. Various attempts have been made to establish SF2 thresholds for radiosensitivity-based grouping of cell lines. In the study by Bentzen (12), the SF2 cutoff value was proposed to screen for patients overreacting to radiotherapy. This was, however, based on the SF2 values generated from a log-normal distribution. In the study by Story et al. (13), 90 skin fibroblast cell lines were clustered into RS, NS, and RR subsets with the k-means algorithm. Nevertheless, no widely used SF2 threshold exists.

Building a systems-biology model to predict SF2 is another approach to identifying radiosensitivity-associated biomarkers (14). This method allows for using SF2 as a continuous dependent variable, reducing the risk that some information carried by SF2 is omitted due to categorization. In 2005, Torres-Roca et al. (15) proposed a gene expression-based model for the prediction of radiosensitivity characterized by SF2 in tumor cell lines. The classifier included a significance analysis of microarrays (SAM) gene selection and multivariate linear regression model. In 2014, Zhang et al. (16) improved the quality of SF2 prediction with their nonlinear approach that consisted of SAM and support vector machine (SVM) regression. In that model, gene expressions of tumor cell strains were used. In 2020, He et al. (17) provided multiple genomic data fused partial least squares deep regression method (MGPLS) for SF2 prediction. In this study, both gene expression and CNV of cancer cell lines were considered. All those approaches were based on the entire range of SF2, without any partitioning into smaller RS or RR subsets.

This research aimed to investigate the relationship between the CNV and the sensitivity to ionizing radiation as measured quantitatively by SF2. We intended to avoid early and arbitrary cell strain grouping by treating radiosensitivity as the categorical variable. Instead, we apply a multivariate linear regression for the rough estimation of SF2. The radiosensitivity signature is based on the selection of markers that allow for satisfactory estimation. We also propose a method to optimally partition SF2 into subranges and fit a separate model for each of them. In this manner, we define data-driven SF2 thresholds for sensitive, normal, and resistant radiosensitivity groups. The local piecewise approach, where the analysis is performed for SF2 intervals (segments), reflects the situation when different factors influence the response to irradiation in sensitive and resistant cells. It allows for identifying specific signatures for each radiosensitivity group.

## 2 Materials and methods

### 2.1 Data collection and preprocessing

Complete sets of data for 129 (55 males and 74 females) non-transformed fibroblast cell strains were available for this study from our cell line collections previously established from normal individuals (6). The institutional review board (IRB) at King Faisal Specialist Hospital and Research Centre has approved the study (RAC-2120-003). Donors have voluntarily participated and signed informed consent. Cells were maintained in a DMEM culture medium supplemented with 15% fetal bovine serum, 100 units/ml penicillin, and 0.1 mg/ml streptomycin and incubated at 37°C in 5% CO_2_ humidified atmosphere. Cellular radiosensitivity measurement using the clonogenic survival curves and the determination of the SF2 was described previously (6). Briefly, contact-inhibited (about 90% in G0-G1) cultures were used, tested cells were trypsinized, counted, and seeded to yield at least 50 colonies at different radiation doses (ranging from 0 to 4 Gy) in each of three replicated flasks, and colonies with at least 50 cells were scored as survivors after incubation for 2-3 weeks and stained with crystal violet. Each cell strain underwent three to five independent experiments. The average plating efficiency was 28% (range 2% - 90%). Survival data were fitted to the linear quadratic model of cell killing [SF = exp (- αD - βD2)], and the surviving fraction at 2 Gy (SF2) was computed and used to characterize the radiosensitivity of each cell strain.

DNA was extracted from non-irradiated fibroblasts using the Puregene DNA Purification Kit (Gentra System, Qiagen, Minneapolis, MN, USA) according to the manufacturer’s instructions. DNA was genotyped using the Affymetrix CytoScan HD microarrays (Affymetrix, ThermoFisher Scientific, Waltham, MA, USA) according to the manufacturer’s instructions as described elsewhere (18, 19). Preprocessing and quality control were performed with Chromosome Analysis Suite 3.1 tool (Thermo Fisher Scientific Inc., Waltham, Massachusetts, USA). The created data set was deposited in the GEO database (http://www.ncbi.nlm.nih.gov/geo/) as the GSE231621 series.

The CNV was obtained for each sample. Fold Change (FC) value represents the sample CNV compared to the Affymetrix reference genome. FC is the ratio of sample and reference signals on a base-2 logarithmic scale, as given in the equation:


$$FC=\log_{2}\frac{sample}{reference}$$

Thus, positive FC values indicate increased CNV in comparison to the reference genome. On the contrary, negative FC corresponds to a genomic loss. We used biomaRt R package to map markers to genes and regulatory features based on the marker chromosomal start position (20, 21). We considered only markers located at autosomes to reduce the risk of sex-related bias and imbalance in cell strain groups identified in further steps. The coverage of autosomal markers provided by Affymetrix CytoScan HD microarrays equals 2,491,915.

### 2.2 Correlation analysis

We aimed to avoid treating cell radiosensitivity as a binary feature. Hence, SF2 served as a continuous measure instead of being resolved into only a base for individuals’ categorization. To investigate the association between radiosensitivity and CNV, the Pearson and Spearman rank correlation coefficients were calculated between SF2 and FC for each marker. All correlation coefficients were tested for significance. The results were corrected for multiple testing with the Benjamini-Hochberg method (22).

### 2.3 Multivariate linear regression models

We aimed to create a global Multiple Input Single Output (MISO) linear regression model for the rough SF2 estimation, which allowed for treating the radiosensitivity characterization quantitatively rather than qualitatively. The proper linear regression model should include only FC values for markers that provide significant information about SF2. Thus, selected features are probably associated with cellular radiosensitivity. Consequently, apart from the SF2 prediction, the model should allow for the radiosensitivity signature identification based on the list of markers that will make the satisfactory prediction possible.

To assess models’ quality, we kept randomly chosen 13 cell strains as a validation set. The remaining 116 cell strains served as a training set for model building. The validation set was selected in a balanced manner so that the included cell lines were distributed equally in the entire SF2 range. For this set only, we tested the Pearson correlation between each FC and SF2. Only the Pearson correlation coefficient was considered because it corresponds to the linear association. The set of potential features for every model consisted only of markers with the strongest correlation between FC and SF2 (p<10^-5^). The rest was rejected as their contribution to the model cannot be significant when the correlation between FC and SF2 is weak.

Each MISO linear regression model was built with the forward feature selection method with Bayes Factor (BF) as a criterion (23). A new feature was added based on the highest BF among all models considered in each step. The model was expanded until BF decreased below 10. This threshold is interpreted as at least strong evidence in favor of the more complicated model (24). The number of features in the model must have been at least 10 times higher than the number of cell strains used for fitting. Hence, for the whole training set, the MISO regression model could maximally consist of 11 features.

We estimated the quality of each model as the Mean Relative Error (MRE), with the Relative Error (RE) for the *i-th* cell strain described by the formula:


$$RE_{i}=\frac{\left|SF2_{predicted_{i}}-SF2_{observed_{i}}\right|}{\left|SF2_{observed_{i}}\right|}$$

We created the MISO linear regression model for the entire training set that covered the whole range of SF2 values.

### 2.4 Piecewise linear regression

We suspect that the relation between SF2 and FC for many markers may be non-constant for the entire range of SF2. Following the SF2 estimation with the global MISO model, we investigated whether fitting different MISO linear regression models in SF2 intervals instead of the whole range would be more accurate. For this reason, we adapted the piecewise linear regression method so that the dependent variable is partitioned instead of the independent one. Hence, we divided cell strains into segments of similar SF2 values and created a separate local MISO linear regression model for each batch in the manner described above. This approach enables fine-tuning radiosensitivity estimation by choosing specific markers for each SF2 subrange. Thus, it allows investigating whether a response to irradiation is associated with different features in cells varying in their sensitivity, which, as we assumed, may represent diverse biological background.

We fitted separate models for two training set subgroups. We used the training set’s SF2 mean value for the division, as it is a threshold typically applied for the categorization of cell strains in similar studies (6, 25). Moreover, we also examined other breakpoints as well as different numbers of segments.

### 2.5 Segment identification by dynamic programming

As stated above, SF2 mean- or median-based segment division is arbitrary and does not separate sensitive and resistant cell strains the best way. To verify whether the different breakpoint choice improves prediction, we implemented a dynamic programming (DP) (26) algorithm to identify the optimal segments (batches). Cell strains sorted according to SF2 were partitioned into segments in a manner to minimize the overall (weighted) MRE for the training set. The overall MRE is an arithmetic mean of relative errors for each cell strain across all batches. It also represents the weighted mean of batch MREs, with weights defined as a proportion of observations assigned to each batch. The overall MRE is given by the formula, where *k* is the segment index from 1 to *K*, and *i* is the cell strain index from 1 to *N*.


$$MRE_{overall}=\;\sum_{k=1}^{K}\left(MRE_{k}\cdot \frac{n_{k}}{N}\right)=\;\frac{\sum_{i=1}^{N}RE_{i}}{N}$$

Moreover, we examined if SF2 partitioning into a higher number of batches increases the quality and perhaps gives a more comprehensive insight into radiosensitivity signature. The selection of the utmost segments was critical as they correspond to the most RS and RR cells. It is their comparison in terms of the CNVs that seems to be crucial for the study. We thus aimed to identify optimal data-driven SF2 breakpoints, as well as the number of segments that allow for the selection of well-established outer batches. However, the number of segments should not be too high to avoid having multiple overfitted models for too few cell strains. Thus, we defined the minimal batch size as 10. This value reflects the requirement for the model to include at least 10 observations per one independent variable.

### 2.6 Comparative analysis

Comparative analysis was applied to determine markers differing in their CNV between the RS and RR groups of cell strains. Due to the large number of comparisons (n=2,491,915), the Mann-Whitney U test was conducted with the significance level set to 10^-5^. Differences between groups were also assessed with Glass rank-biserial correlation (27), which is a non-parametric effect size estimate. Interpretation of its absolute values is defined as small, medium, large, and very large for the following thresholds: 0.1, 0.3, 0.5, and 0.7, respectively (28, 29).

## 3 Results

### 3.1 Surviving fraction after irradiation

**Figure 1:**
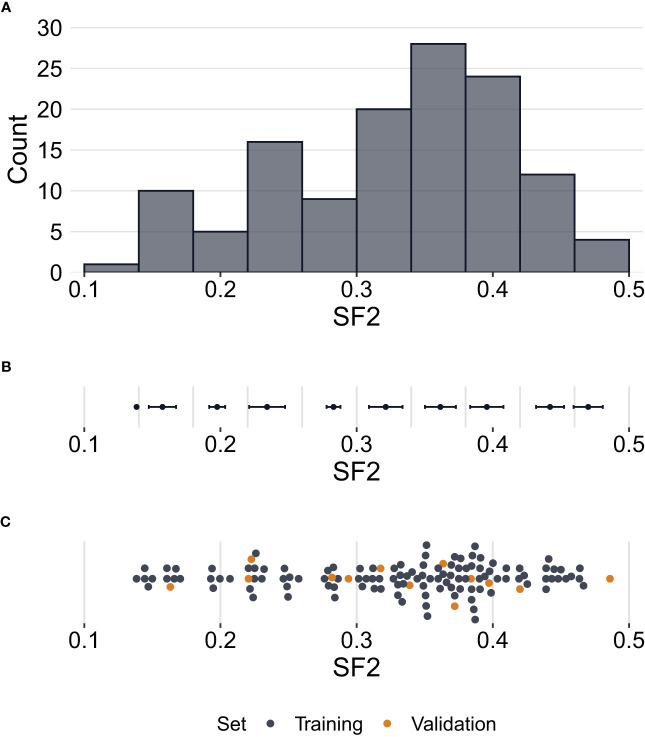
Distribution of surviving fraction at 2 Gy (SF2) values. **(A)** – Histogram showing counts of SF2 values for all cell strains (N=129) in the study. **(B)** – Error bars per histogram bin representing SF2 mean values ± standard deviation. **(C)** – Distribution of SF2 values indicating training (n=116) and validation (n=13) sets. Each point corresponds to one cell strain. Some points are vertically shifted to avoid overlapping.

Radiosensitivity measured by the SF2 ranged between 0.1384 and 0.4860, with a mean of 0.3273 and a median of 0.3490. The standard deviation of SF2 equalled 0.0868, while the interquartile range was 0.1105. The U Mann-Whitney test showed no significant differences in SF2 between males and females (p=0.2521). The SF2 distribution is presented in **Figure 1**.

### 3.2 Association between radiosensitivity and copy number variation

**Figure 2:**
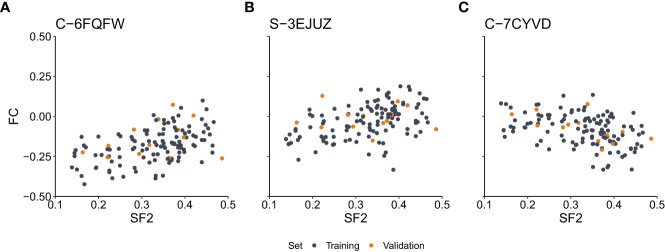
Scatterplots of the surviving fraction at 2Gy (SF2) and fold change (FC) values for the exemplary markers with the significant Spearman rank correlation. **(A)** – Marker C-6FQFW located in the *GABRA2* gene intron. **(B)** – Marker S-3EJUZ located in the *MS4A6A* gene exon. **(C)** – Marker C-7CYVD located in the *FAT4* gene intron. Data points correspond to cell strains in the training and validation sets as indicated.

We assessed the correlation between SF2 and FC for each marker. We observed a medium effect (|r|>0.3) for 7,889 and 7,670 markers based on the Pearson and Spearman correlation coefficients, respectively. However, after the Benjamini-Hochberg correction for multiple testing, no result remained significant for the Pearson correlation. As for the Spearman rank correlation coefficient, the association was significant for nine markers (S-3YLSD, C-7KGRE, C-6ZIYI, C-6FQFW, C-7CYVD, S-3EJUZ, C-5XHWW, C-3EZUM, C-3KFRW) (**Supplementary Table 1**), three with negative correlation (C-7KGRE, C-7CYVD, C-5XHWW), for which the rise in the copy-number is associated with increased radiosensitivity, and six with positive correlation, for which the drop in the copy-number is correlated with increased radiosensitivity. The six markers are located in the introns (*GABRA2*, *FAT4*, *RBFOX1*, *RRN3*, and *PDXDC1* genes), exons (*MS4A6A* gene), or 3’-UTR regions (*RRN3*, *PDXDC1* genes). The remaining three markers are located in the intergenic regions at chromosomes 2 and 17. The scatterplots of the SF2 versus FC for three exemplary markers are presented in **Figure 2**.

### 3.3 Global multivariate linear regression model

The division into training and validation sets is presented in **Figure 1**. In total, the p-values of the Pearson correlation significance test were lower than 10^-5^ for 299 markers. Those markers were selected as the potential features for all linear regression models built in the study.

The final global MISO regression model fitted based on the entire training set consisted of ten markers, seven of which were mapped to gene regions (**Table 1**). Moreover, two markers were in regions with regulatory potential (**Table 1**). MRE for this model was equal to 0.1142 and 0.2253 for the training and validation sets, respectively.

**Table 1:** Linear regression parameters with gene and functional annotations. All cell strains in the training set (n=116) were used for fitting and the entire SF2 range was considered.

Marker	Gene	Genomic region	Regulatory feature	Regression coefficient	P-value
Intercept	–	–	–	0.3460	<0.0001
S-4LQMA	DCC	Intron	–	-0.0834	0.1917
C-7NZOT	KAZN	Intron	Binding Motifs: ENSM00894371323, TEAD4::SOX15; ENSM00894371323, POU2F1::DLX2	0.0573	0.0003
C-4DHPL	–	Intergenic	–	-0.2337	<0.0001
C-6FQFW	GABRA2	Intron	–	0.1743	0.0001
C-5ZTNW	PHF14	Intron	–	-0.1602	0.0005
C-7KDQH	LINC01090	Intron	–	-0.2120	0.0001
S-3SHPD	TSNAX-DISC1, DISC1	Intron, Intron	–	0.1700	0.0018
C-5TMHZ	–	Intergenic	–	-0.1227	0.0005
S-4CYFP	–	Intergenic	–	0.1295	0.0007
S-3LRAO	CTD-3012A18.1, RP11-460B17.3	Intron, Intron	Promoter Flanking Region: ENSR00001533279 Binding Motif: ENSM01015619192, CUX1::NHLH1	-0.1509	0.0021

### 3.4 Local piecewise multivariate linear regression models

The first piecewise MISO linear regression attempt was to create separate models for RS and NS groups. That division was based on the mean of SF2 for the training set, which is equal to 0.3273. The RS training set consisted of 46 cell strains, while the NS training set included the remaining 70 cell strains. The model fitted for the RS subrange included four markers, with three of them mapped to the following gene regions: *FAHD2A*, *DCC*, *ISX*, and *RP1-272J12.1*. The model for the NS group consisted of FC for five markers, three of which were located inside the following genes: *PROZ*, *FOXP1-AS1*, *FOXP1*, *CTD-3012A18.1*, and *RP11-460B17.3* (**Supplementary Table 2**). Moreover, binding motifs were also identified for three markers (**Supplementary Table 3**). The overall MRE was better than for the model fitted on the entire training set. However, we observed that the model performance was lower for the RS subrange than for the NS response group (**Table 2**).

**Table 2:** The Mean Relative Errors (MREs) of piecewise MISO linear regression model along with the mean SF2 for the training set serving as the breakpoint.

Cell strains	SF2	MRE	
		Training set	Validation set
NS* group	>0.3273	0.0499	0.0774
RS** group	<0.3273	0.1140	0.3109
**All (weighted MRE)**		0.0753	0.1851

*NS, normally sensitive to irradiation; **RS, radio-sensitive.

**Figure 3:**
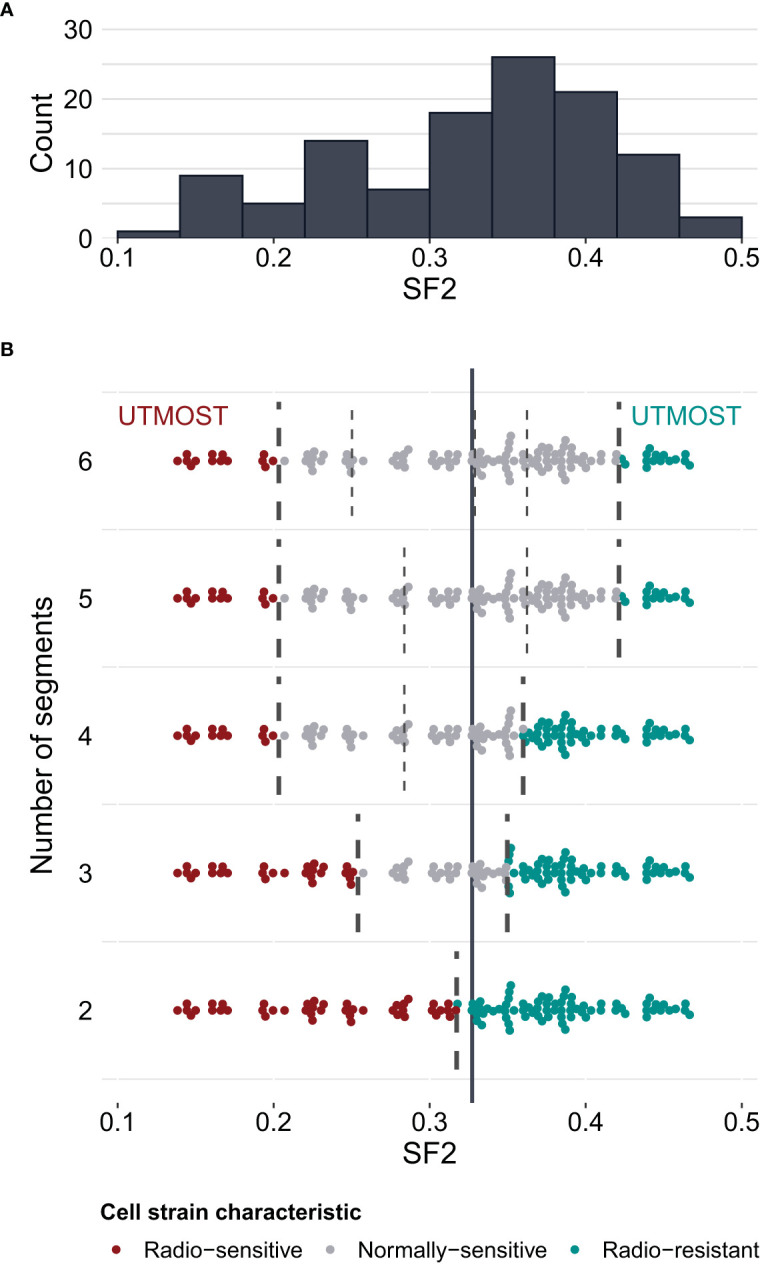
Survival fraction at 2 Gy (SF2) partitioning for piecewise MISO linear regression obtained with the dynamic programming (DP) algorithm. **(A)** – distribution of SF2 values in the training set of cell strains (N=116). **(B)** – division of SF2 values into 2 to 6 subranges (segments). Breakpoints are marked with broken lines. The solid vertical line marks the mean SF2 in the training set (equal to 0.3273). The utmost segments are highlighted for each division: the red data points correspond to the radio-sensitive (RS) cell strains and the green data points correspond to the radio-resistant (RR) cell strains. The remaining gray data points were categorized as having a normal sensitivity (NS), i.e. expressing regular response to irradiation. Some points are vertically shifted to avoid overlapping.

To select optimal breakpoints for segmented regression, we fitted the models with the dynamic programming (DP) approach. The obtained SF2 breakpoints and the overall MRE values for different numbers of segments are presented in **Table 3**. The MREs calculated separately for each subrange can be found in **Supplementary Table 4**. The MRE decreases as the number of batches increases. However, the borders of the utmost segments stabilized after partitioning SF2 into five subranges (**Figure 3**). Thus, the MISO regression model with five batches was assumed to provide an adequate division into segments and a satisfactory section of the markers associated with the radiation response within each subgroup. Moreover, the utmost breakpoints served as thresholds to identify the most RS and RR cell strains.

**Table 3:** The weighted Mean Relative Errors (MREs) of piecewise MISO linear regression models along with the SF2 breakpoints defined with the dynamic programming (DP) algorithm.

Number of segments	SF2 breakpoints*	Weighted MRE	
		Training set	Validation set
2	0.3168	0.0712	0.1828
3	0.2508/0.3490	0.0488	0.1093
4	0.1996/0.2835/0.3599	0.0356	0.1044
5	0.1996/0.2835/0.3613/0.4197	0.0275	0.0812
6	0.1996/0.2496/0.3276/0.3613/0.4197	0.0222	0.0686

* The breakpoint was defined as the highest SF2 value among the cell strains included in the given segment as determined by the DP algorithm.

**Figure 4:**
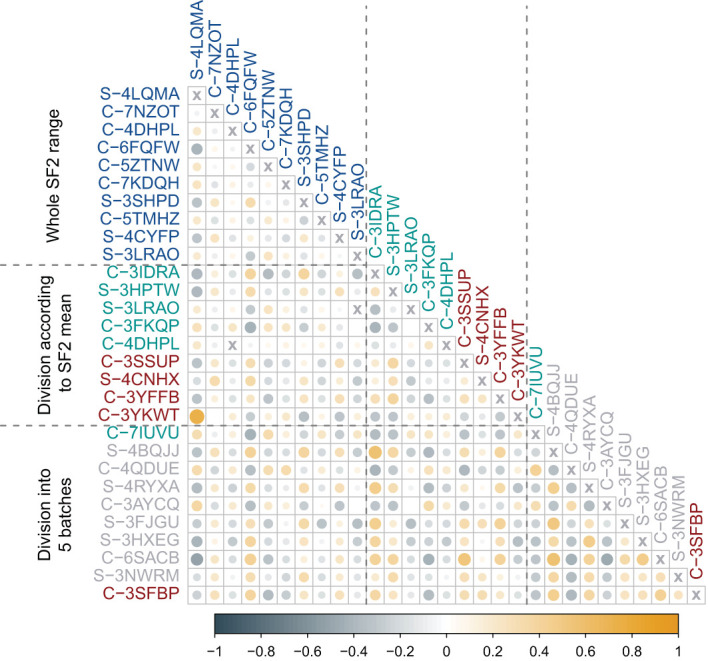
Pearson correlation coefficient (*r*) between fold changes (FCs) of markers identified in various approaches. The first block of markers (blue) corresponds to the multivariate linear regression model fitted for the entire range of SF2 values. The second block includes markers used in two separate models for cell strains with SF2 lower (red) or higher (green) than the mean. The third block corresponds to piecewise multivariate linear regression with SF2 partitioning into five segments with the dynamic programming (DP) algorithm. Markers included in models for utmost segments are marked with green (radio-resistant RR cell strains) and red (radio-sensitive RS cell strains). Markers used in the remaining three models are marked in gray (normally sensitive NS, i.e. having regular response). The color of circles represents the direction of association (steel blue corresponds to negative Pearson correlation coefficient (*r*) values, while orange to positive ones). The color intensity and circle size reflect the strength of the correlation.

The selected piecewise linear regression model with five segments included one marker for each utmost batch corresponding to the most RS and the most RR cell strains (**Table 4**). Both those markers (C-3SFBP and C-7IUVU, respectively) were mapped to gene regions: *MCC* in the RS group and *SLC1A6* in the RR group, respectively (**Table 4**). In addition, the C-3SFBP marker used in the low SF2 local model was also located in the enhancer region (ENSR00001697205). Furthermore, 18 binding motifs were found in this genomic position, including those associated with the evolutionarily conserved RFX transcription factor family (**Supplementary Table 5**). We assessed the pairwise association between FC of markers included in various local models with the Pearson correlation coefficient (**Figure 4**).

**Table 4:** Parameters with gene and functional annotations for piecewise MISO linear regression model.

Batch ID	Marker	Gene name	Genomic region	Regulatory feature	Regression coefficient	P-value
**1**	Intercept	–	–	–	0.4412	<0.0001
	C-7IUVU	SLC1A6	Intron	–	-0.1035	0.0017
**2**	Intercept	–	–	–	0.3776	<0.0001
	S-4BQJJ	–	Intergenic	Enhancer (ENSR00001567932)	0.0780	<0.0001
	C-4QDUE	–	Intergenic	–	0.1105	<0.0001
	S-4RYXA	–	Intergenic	–	0.0835	0.0001
**3**	Intercept	–	–	–	0.3285	<0.0001
	C-3AYCQ	BBS9	Intron	–	-0.1305	0.0002
	S-3FJGU	ST6GALNAC3	Intron	–	-0.0934	0.0019
	S-3HXEG	–	Intergenic	Promoter Flanking Region (ENSR00001504317)	0.0744	0.0055
**4**	Intercept	–	–	–	0.2790	<0.0001
	C-6SACB	–	Intergenic	–	0.1293	<0.0001
	S-3NWRM	ROBO1	Intron	–	0.1330	0.0033
**5**	Intercept	–	–	–	0.1713	<0.0001
	C-3SFBP	MCC	Intron	Enhancer (ENSR00001697205)	0.1821	<0.0001

SF2 values were partitioned into 5 segments (batches) numbered from 1 to 5. The first segment includes the most resistant cell strains (for which SF2 is the highest). The fifth segment includes the most sensitive cell strains (for which SF2 is the lowest).

### 3.5 Comparison of most sensitive and most resistant cell strains

**Figure 5:**
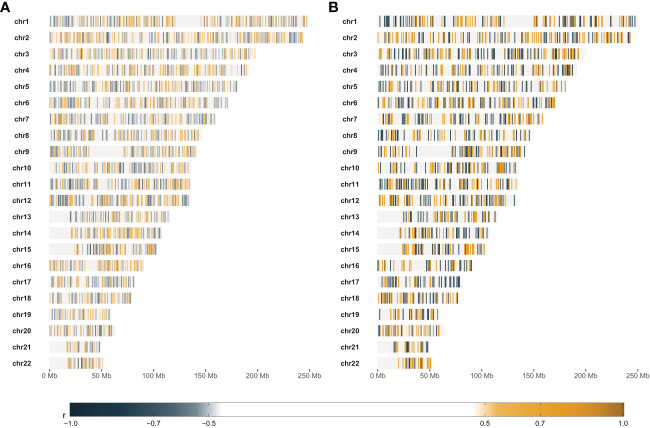
Results of comparative statistical analysis between radio-sensitive (RS) and radio-resistant (RR) cell strains. **(A)** – Genomic positions of markers with at least large Glass rank-biserial correlation (r) effect. RS and RR cell strains were selected based on the lower and upper quartile SF2, respectively. **(B)** – Genomic positions of markers with at least very large Glass rank-biserial correlation (r) effect. RS and RR cell strains were selected as the outer batches in the dynamic programming-based piecewise linear regression model. Positive Glass rank-biserial correlation (marked in orange) means that the CNV in the RR cell strains is higher than in the RS ones. On the contrary, negative Glass rank-biserial correlation (marked in steel blue) means that CNV in RR cell strains is lower than in RS ones.

**Figure 6:**
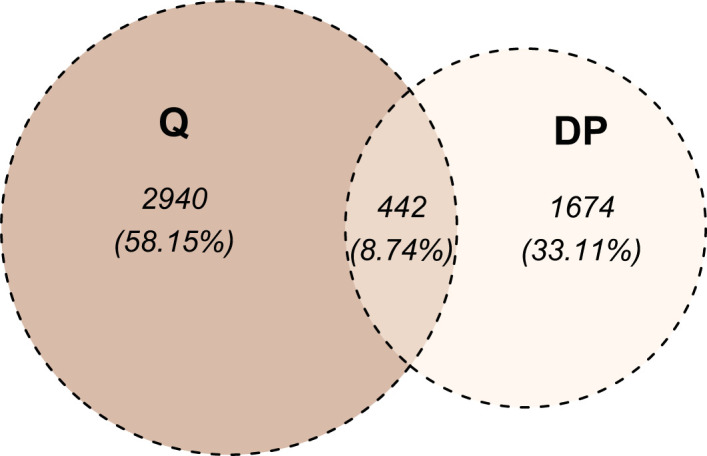
Venn diagram of markers differentiating radio-sensitive (RS) and radio-resistant (RR) cell strains. The division into RS and RR categories was based on SF2 quartiles (denoted as Q) or segments identified with the dynamic programming algorithm (denoted as DP). For the Q-based approach, the at least large effect defined the differentiating markers, while for the DP-based approach, at least a very large effect was required.

We selected the most RS and the most RR cell strains with two approaches: based on piecewise regression breakpoints (DP-based) and quartile values (Q-based). CNVs of the most RS and the most RR cell strains were compared with the Mann-Whitney U test. In both cases (DP- and Q-based), we detected no statistically significant differences after the Benjamini-Hochberg correction for multiple testing due to a large number of comparisons (n=2,491,915). However, Mann-Whitney U test p-values were lower than 10^-5^ for 39 and 110 markers in DP-based and Q-based approaches, respectively. The observed Glass rank-biserial correlation effect was at least large for 3,382 markers in the Q-based comparison (**Figure 5A**). In total, 2,224 introns, 142 exons, 66 3’-UTRs, and ten 5’-UTRs were identified within those markers. For the Q-based approach, only two markers had a very large effect: one located in the *GABRA2* gene and the other in the intergenic sequence. No regulatory functions were identified for both of those markers. Comparatively, at least a very large effect was observed for 2,116 markers in the DP-based variant model (**Figure 5B**). Those markers were mapped to 1383 intron regions, 78 exon regions, 42 3’-UTR regions, and seven 5’-UTR regions in total. For both approaches, genomic positions of differentiating markers were distributed uniformly across all chromosomes (**Figures 5A, B**). There were only 442 (8.74%) common markers identified in both Q- and DP-based comparisons (**Figure 6**).

## 4 Discussion

Inter-individual variations in radiosensitivity are well recognized and impact not only medical radiology patients but also the risks of radiation exposure in the human population at large (30). Efforts to develop quantitative methods to better measure radiosensitivity have been ongoing for decades with stagnating advancements. It is, therefore, not surprising that applications of biological endpoints and mathematical modeling have played prominent roles in the characterization of radiosensitivity in cell lines *in vitro* and in radiotherapy patients *in vivo*. In general, to model the relationship between SF2 and FC, an assumption needs to be made about its shape for each marker, which was a challenge given the large number of markers and the unknown optimal number of SF2 subranges. We overcome these challenges by using a piecewise multivariate linear regression with a dynamic programming approach. Furthermore, subdividing SF2 into subranges and fitting separate models not only improved the prediction of SF2, but also made it safe to assume that the relationship between FC and SF2 is locally linear. In this study, we put forth a multivariate piecewise linear regression model to categorize cell strains based on their radiosensitivity and group-wisely investigate its association with the CNVs obtained from GWAS experiments. Fitting local models allows us to identify CNV markers specific for cell strains similar in their radiation response, in particular for radio-sensitive and radio-resistant. Hence, we potentially address the challenge of adjusting the radiotherapy not only to over- but also under-responders.

For the global MISO linear regression model fitted for the entire SF2 range, the MREs were 0.1142 and 0.2253 for the training and validation sets, respectively. The root-mean-square error (RMSE) values were equal to 0.0405 and 0.0879 for the training and validation sets, respectively. These results are smaller than 0.094 and 0.16 reported by He et al. (17) and Zhang et al. (16), respectively, which were achieved in their linear approaches. Their nonlinear models performed better, with RMSE equal to 0.0025 and 0.011, respectively. Nevertheless, despite high MRE in the validation set (0.2253), our global linear regression model provided the selection of markers associated with SF2 in the whole SF2 range. The Spearman rank correlation between FC and the whole SF2 range was significant for nine CNV markers (S-3YLSD, C-7KGRE, C-6ZIYI, C-6FQFW, C-7CYVD, S-3EJUZ, C-5XHWW, C-3EZUM, C-3KFRW). Although the results showed that radiosensitivity could be associated with loss and gain in CNVs, for most of those markers, the correlation between SF2 and FC was positive, meaning the increased radiosensitivity was associated with a drop in the copy number. Using Affymetrix 6.0 SNP arrays to survey common CNVs in a cohort of 50 RS lymphoblastoid cell lines, Li et al. (31) reported a dominance of chromosomal gains over losses, which the authors deemed as inconsistent with the traditional concept of the molecular basis of RS. Nevertheless, the various findings of the CNV studies enrich the molecular mechanisms of RS by highlighting that chromosomal loss and gain may be an important pathway in regulating the radiosensitivity phenotype.

Dividing the cell strains based on the mean SF2 and fitting two separate linear regression models improved the SF2 estimation. Weighted MRE for both training and validation sets was lower than for the model built for the entire SF2 range (**Table 2**). Moreover, we observed that MRE values were lower for the RR subrange than for the RS one. Hence, SF2 could be estimated more accurately in its high values. The satisfactory prediction was harder to obtain for more RS cell strains also in other studies. All models proposed by He et al. (17), Zhang et al. (16), and Torres-Roca et al. (15) perform more poorly for low SF2 values, corresponding to RS cell strains. What is more, RR and RS models differ in the selected markers, which suggests that various factors influence the response to irradiation in sensitive and resistant cells. This conclusion and the improvement of performance due to SF2 partitioning supports the decision to use segmented linear regression for the local identification of CNV associated with sensitivity. Due to the difficulties in the prediction of low SF2, we decided to use MRE for the model quality estimation. The relative error does not discriminate against the wrong estimation for RS cell strains, while RMSE does. If the observed SF equals 0.1 and the predicted SF2 is two times higher, the impact on the RMSE value is the same as when the predicted SF2 equals 0.8 instead of 0.7.

We compared the weighted MREs of two two-segment models: the first one with the breakpoint defined by mean SF2 (0.0753 and 0.1851 for the training and validation sets, respectively; **Table 2**), and the second one with a breakpoint obtained with DP approach (0.0712 and 0.1828 for the training and validation sets, respectively; **Table 3**). Even though SF2 thresholds used for splitting in both approaches were very similar (the mean 0.3273 compared to 0.3168), there was a slight decrease in the overall MRE. Thus, the data-driven SF2 breakpoint increased prediction quality. Weighted MRE decreased as the number of segments increased (**Table 3**). The border for the RS segments remained constant for the number of batches from 4 to 6 (**Figure 3**). For 5 and 6 batches, the thresholds for both utmost salient segments stay the same. Hence, we chose the division into 5 batches as the optimal solution. For this local MISO linear regression model, RMSE equalled 0.0107 and 0.0279 for the training and validation sets, respectively. The prediction is thus similar to Zhang et al. (16) nonlinear approach but worse than for the nonlinear model proposed by He et al. (17). Both RMSE and MRE decreased substantially in comparison with our global model for the entire SF2 range. Moreover, the segmented regression approach provided separate markers related to sensitivity to radiation in various specific SF2 intervals.

The highest SF2 included in the RS batch was 0.1996. This value is similar to the cutoff SF2 equal to 0.19 for over-responders screening proposed by Bentzen (12). With the k-means approach, Story et al. (13) suggested cell strains with SF2<0.26 to be RS and cell strains with SF2>0.36 to be RR. Those thresholds are in agreement with the ones we obtained for three batches (SF2 ≤ 0.2508 and SF2>0.3490) and four batches (the highest SF2 in the RS group equaled 0.1996 and the lowest SF2 in the RR group equaled 0.3599).

The most RS and RR groups defined based on the DP approach were more extreme in their response and less numerous than the categories obtained with the SF2 quartiles. Those factors led to an increased effect size (as determined by the Glass rank-biserial correlation) so that we identified more markers with at least large and very large effects (**Figures 5**, **6**). The Glass rank-biserial correlation was more appropriate for the comparative analysis than the Mann-Whitney U test due to a large number of markers. Since the number of comparisons was very high, corrections for multiple testing gave insignificant results. Nevertheless, at least a very large effect was detected for 2,116 markers of differential radiosensitivity in DP-based approach compared to 3,382 markers with at least large effect in the Q-based model. Only 442 (8.74%) markers were common in both comparisons (**Figure 6**). Differentiating markers were mapped to various genomic regions including introns, exons, 3’- and 5’-UTR regions and were distributed uniformly across all chromosomes (**Supplementary Tables 6** and **7** for Q-based and DP-based approach, respectively).

The data used in this study was collected using Affymetrix CytoScan HD microarrays, which provide measurements for nearly 2.7 million markers, including 2,491,915 autosomal markers. They are distributed across the genome, hence many of the measured markers were located in introns or intergenic regions. Consequently, most of the potential predictive features for our models were intergenic or intron. However, many of these intergenic/intron markers were annotated with regulatory regions such as enhancers or potential transcription factor binding motifs (see **Table 1**). This suggests that alterations in these fragments may indirectly (through regulation of gene expression) affect various cellular processes, related to response to stress and irradiation.

Although the marker identification described here requires biological validation to ascertain the involvement of the genes associated with the CNV markers, it is beyond the scope of the present report. Nevertheless, evidence of mechanistic involvement is mounting from studies in radiotherapy patients. A CNV analysis in prostate cancer patients has identified seven genomic regions associated with proctitis following radiotherapy (32). However, there are still limitations in CNV studies mainly related to small sample size, few populations studied, and the potential transitional nature of copy number signatures due to the diversity of mutational processes that give rise to these alterations (33). Therefore, larger studies in multiple populations are required to validate these results particularly in radiotherapy patients in a similar way to the multi-centers radiogenomic consortium that had identified genetic variants associated with radiation toxicities in prostate cancer patients following radiation therapy (34). Furthermore, many other non-genomic assays (such as DNA repair, cell death, proliferation, hypoxia, etc.) had been tested and found to be variably correlated with cellular, clinical radiosensitivity, and potentially susceptibility to radiation-induced cancer (4).

Indeed, different cell types and tissues could have distinct biomarker profiles that influence their radiation responses. As such, any potential clinical application of our findings would need to be integrated with a broader range of factors, including tissue-specific considerations and other relevant clinical data. Nevertheless, the findings of this study is a step forward and may have important clinical implications for individualizing radiotherapy of cancer patients. Once validated and applied in clinics, this approach could potentially enable the stratification of radiotherapy patients into groups of mild, moderate, and severe responders. This information can help clinicians determine the optimal dose of radiation for each patient to minimize complications and improve outcomes. This would greatly simplify and streamline the process of individualizing radiotherapy for cancer patients and estimate the risks of radio-susceptibility of exposed individuals. Eventually, this approach has the potential to improve patient outcomes and minimize the risks associated with radiation exposure.

## 5 Conclusions

In this study, we described a machine learning data-driven method for the classification of sensitive, normal, and resistant radiation responders. We created the global and local multivariate linear regression models using the CNV measurements. Feature selection while fitting the model produced the set of CNV markers associated with cellular radiosensitivity for both: the whole investigated SF2 range and the smaller intervals of interest, with the use of the dynamic programming algorithm. Notably, SF2 partitioning not only improves the SF2 prediction but also provides separate narrow lists of radiosensitivity-related features in cells varying in their radiation response. Ultimately, segment-related markers, along with potential tissues’ specificities and clinical data, can be used to identify radiotherapy patients who are most sensitive and require reduced dose to avoid normal tissue complications and the most resistant, eligible for dose escalation to improve local tumor control.

## Data availability statement

The datasets presented in this study can be found in online repositories. The names of the repository/repositories and accession number(s) can be found in the article/**Supplementary Material**.

## Ethics statement

The institutional review board (IRB) at King Faisal Specialist Hospital and Research Centre has approved the study (RAC-2120-003). Donors have voluntarily participated and signed an informed consent.

## Author contributions

Conceived of designed study: GA and JP. Performed research: JT, SM, SB, and NA-H. Analyzed data: JT and JP. Contributed new methods or models: JT, JP, and SM. Wrote the paper: JT and GA. Editing: GA and JP. Review: All authors. All authors contributed to the article and approved the submitted version.
